# Production of *Gloeophyllum trabeum* Endoglucanase Cel12A in *Nicotiana benthamiana* for Cellulose Degradation

**DOI:** 10.3389/fpls.2021.696199

**Published:** 2021-06-28

**Authors:** Kyoung Rok Geem, Younho Song, Inhwan Hwang, Hyeun-Jong Bae, Dong Wook Lee

**Affiliations:** ^1^Department of Bioenergy Science and Technology, Chonnam National University, Gwangju, South Korea; ^2^Bio-Energy Research Center, Chonnam National University, Gwangju, South Korea; ^3^Department of Life Sciences, Pohang University of Science and Technology, Pohang, South Korea; ^4^Department of Integrative Food, Bioscience and Biotechnology, Chonnam National University, Gwangju, South Korea

**Keywords:** cellulase, GtCel12A, *Nicotiana bentamiana*, molecular farming, protein solubility, endoglucanase activity

## Abstract

Lignocellulosic biomass from plants has been used as a biofuel source and the potent acidic endoglucanase GtCel12A has been isolated from *Gloeophyllum trabeum*, a filamentous fungus. In this study, we established a plant-based platform for the production of active GtCel12A fused to family 3 cellulose-binding module (CBM3). We used the signal sequence of binding immunoglobulin protein (BiP) and the endoplasmic reticulum (ER) retention signal for the accumulation of the produced GtCel12A in the ER. To achieve enhanced enzyme expression, we incorporated the M-domain of the human receptor-type tyrosine-protein phosphatase C into the construct. In addition, to enable the removal of N-terminal domains that are not necessary after protein expression, we further incorporated the cleavage site of *Brachypodium distachyon* small ubiquitin-like modifier. The GtCel12A-CBM3 fusion protein produced in the leaves of *Nicotiana benthamiana* exhibited not only high solubility but also efficient endoglucanase activity on the carboxymethyl cellulose substrate as determined by 3,5-dinitrosalicylic acid assay. The endoglucanase activity of GtCel12A-CBM3 was maintained even when immobilized on microcrystalline cellulose beads. Taken together, these results indicate that GtCel12A endoglucanase produced in plants might be used to provide monomeric sugars from lignocellulosic biomass for bioethanol production.

## Introduction

Due to the energy crisis caused by the gradual depletion of fossil fuel reserves and the accumulation of greenhouse gases such as carbon dioxide and methane, caused by high consumption of fossil fuels, the demand for alternative renewable energy sources is increasing (Jeswani et al., 2020; Kumar et al., 2020; Liu et al., 2021). Alternative energy sources include solar, wind, geothermal, tidal, and hydroelectric energy along with bioenergy. Among them, bioenergy can be produced from living or once-living organisms (biomass) through a variety of biomass conversion and biorefinery technologies (Cho et al., 2020; Srivastava et al., 2021). Among the several types of biomass resources, plant-derived lignocellulosic biomass is the most abundant raw material (Fatma et al., 2018; Toor et al., 2020). Lignocellulose is mainly composed of carbohydrate polymers, such as cellulose and hemicellulose, and an aromatic polymer, lignin. During lignocellulosic biomass-derived biofuel production, the efficient conversion of carbohydrate polymers into monomeric sugars, later used for ethanol production through fermentation, are of utmost importance for cost-efficiency (Liu et al., 2018; Ali et al., 2020; Barbosa et al., 2020). Moreover, the production costs of cellulase, an enzyme converting carbohydrate polymers into monomeric sugars, account for ~40% of the total lignocellulose-based bioethanol production cost (Behera and Ray, 2016). Thus, the development of a platform for the cost-efficient production of cellulase harboring high stability and activity might provide a promising avenue in the bioethanol industry.

Cellulases are glycoside hydrolases that catalyze the hydrolysis of β-1,4-glycosidic linkages in cellulose polymers. Cellulase is mainly composed of a catalytic domain that cleaves the glycosidic bond and a carbohydrate-binding module that binds to the substrate, thereby guiding the catalytic domain to the polysaccharide chains. According to the structure and mode of action, cellulases can be categorized into at least three groups - endoglucanases, exoglucanases, and β-glucosidases. The mechanisms underlying the function of these enzymes in cellulose decomposition have been well-described in previous reviews (Obeng et al., 2017; Barbosa et al., 2020). Endoglucanases (EC 3.2.1.4) are enzymes that catalyze the internal cleavages of β-glycosidic bonds in cellulose, thus releasing short polysaccharides that are further degraded by β-glucosidases or cellobiases. Many industrial cellulase enzymes have been sourced from various organisms - mainly fungi and bacteria (Obeng et al., 2017). For example, *Trichoderma reesei* filamentous fungus secretes a diverse mixture of cellulases and hemicellulases (Bischof et al., 2016; Obeng et al., 2017); novel strains of *T. reesei* producing high levels of cellulases have been identified through successive strain improvement (Peterson and Nevalainen, 2012). In addition, several industrial cellulases have been produced using different expression platforms including bacteria (Maki et al., 2009), yeast (Oh and Jin, 2020), thermostable fungi (Saroj et al., 2018), insect cell lines (Li et al., 2010), and plants (Jin et al., 2003; Kim et al., 2010; Garvey et al., 2014; Lambertz et al., 2014).

With regard to the production of industrial enzymes such as cellulases, plants exhibit several advantages over other systems such as bacteria, yeasts, and mammalian cells. First, the growth of transgenic plants is highly scalable. Second, the cost for plant growth is relatively lower than that for animal cell culture. Third, plants are almost free of endotoxins such as lipopolysaccharides, which are abundant in bacterial cells. Further, the conditions for plant growth are much less affected by microorganisms that are detrimental to mammalian cell cultures (Buyel et al., 2017; Moon et al., 2019; Muthamilselvan et al., 2019; Knodler and Buyel, 2021; Schillberg and Finnern, 2021). In addition, previous studies indicated that various useful proteins of different origins, such as bacteria, bacteriophage, animals, and red algae, remained functional when produced in tobacco (*Nicotiana benthamiana*) (Garvey et al., 2013; Islam et al., 2019, 2020; Kumari et al., 2020; Razzak et al., 2020). One of the most important considerations in protein production using plants is how target proteins can be expressed in large amounts because the cost-effectiveness of protein production is often highly dependent on the protein amounts yielded from a unit cultivation area. Thus, to enhance the production of foreign proteins, several sequences such as 5′-untranslated region (UTR) and HSP transcriptional terminator have been formulated and incorporated into plant expression vectors (Nagaya et al., 2010; Kim et al., 2014; Islam et al., 2019). To avoid the proteolytic degradation of target proteins in the cytosol, expressed proteins can be sequestered to subcellular organelles such as endoplasmic reticulum (ER) or chloroplasts by incorporating organellar targeting signals that are proteolytically removed by organellar peptidase after organellar targeting of proteins (Lee and Hwang, 2018; Islam et al., 2019; Muthamilselvan et al., 2019; Margolin et al., 2020b). ER is an organelle in which several posttranslational modifications such as glycosylation or disulfide bond formation occur, thereby contributing to the functionality of produced proteins (Margolin et al., 2018, 2020a).

Recently, Oh et al. (2019) isolated and characterized the acidic endoglucanase GtCel12A (*Gloeophyllum trabeum* Cel12A). In this study, it was shown that this enzyme displayed highest activity on β-glucan, followed by lichenan and carboxymethyl cellulose (CMC) and xyloglucan (Miotto et al., 2014; Oh et al., 2019). GtCel12A produced from *Pichia pastoris* displayed not only high enzymatic activity but also synergistic effects in combination with commercial cellulase on hydrogen peroxide-acetic acid-pretreated lignocellulosic biomass (Oh et al., 2019). In this study, we attempted to produce GtCel12A from *N. benthamiana* to establish a plant-based platform for GtCel12A production.

## Materials and Methods

### Plant Materials and Growth Conditions

*N. benthamiana* plants (NCBI:txid4100) were grown in a greenhouse at 23–24°C and 40–65% relative humidity with a 16-h light/8-h dark cycle on soil. The leaves of 6–7-week-old plants were used for agro-infiltration.

### Plasmid DNA Construction

The *GtCel12A* sequence (NCBI, HQ163778) was obtained through gene synthesis (Bioneer corp., Daejeon, Korea). In this study, the sequence corresponding to the N-terminal hydrophobic signal sequence (amino acids 1–20) was deleted from the full-length *GtCel12A*. To generate the *BiP-M-bdSUMO-GtCel12A-CBM3-HDEL* construct, we digested with XmaI and Acc65I the pCambia1300 plant expression vector (Komori et al., 2007; Razzak et al., 2020), containing the sequences encoding for the BiP signal sequence, M domain of the human receptor-type tyrosine-protein phosphatase C, SUMO domain, and CBM3-HDEL, and ligated the *GtCel12A* sequence into it that was digested with the same restriction endonucleases.

### Agro-Infiltration of *BiP-M-bdSUMO-GtCel12A-CBM3-HDEL* or *BiP:bdSENP1:HA* Into the *N. benthamiana* Leaves

The constructs *BiP-M-bdSUMO-GtCel12A-CBM3-HDEL* or *BiP:bdSENP1:HA* (Islam et al., 2020) were transformed into *Agrobacterium tumefaciens* (EHA105). *A. tumefaciens* cells harboring binary vector constructs were introduced into *N. benthamiana* leaves via syringe infiltration as described previously (Islam et al., 2019; Razzak et al., 2020). In every agro-infiltration experiment, *A. tumefaciens* harboring p38, which is derived from *Turnip crinkle virus* and encodes a suppressor of host gene-silencing, was co-infiltrated at OD_600_ value of 0.8 (Qu et al., 2003; Islam et al., 2019).

### Purification of MSC-GtCel12A From the *N. benthamiana* Leaves

Leaves (10 g), harvested at 3, 5, and 7 days after agro-infiltration, were ground in liquid nitrogen. Total protein extracts were prepared using 30 mL of protein extraction buffer (50 mM Tris-HCl pH 7.5, 150 mM NaCl, 1 mM DTT, 1% [v/v] Triton X-100, and 1 X EDTA-free protease inhibitor cocktail (Roche, switzerland). After incubation at 4°C for 15 min, the total protein extracts were filtered through Miracloth (Merck Millipore, USA) to remove debris. Subsequently, 100 μL of protein extracts was collected as total fraction. The protein extracts were then centrifuged at 19,400 × *g* for 15 min, and 100 μL of the supernatant was collected as soluble fraction. The samples in the pellet fraction were resuspended with 30 mL of protein extraction buffer, and 100 μL of samples was collected as pellet fraction. The remaining soluble fraction after centrifugation was used for the purification of BiP-M-bdSUMO-GtCel12A-CBM3-HDEL with microcrystalline cellulose (MCC) beads (Sigma-Aldrich, St. Louis, MO, USA; CAS Number 9004-34-6) as described previously (Islam et al., 2019). Briefly, the remaining soluble fraction was incubated with 1 mL of MCC beads pre-suspended with water at 1:1 ratio at 4°C for 3 h, followed by centrifugation at 115 × *g* for 2 min. Then, 100 μL of the supernatant fraction was collected as unbound fraction. The MCC beads containing bound proteins were washed with wash buffer (containing 50 mM Tris-HCl and 150 mM NaCl; pH 7.5) three times (W1–W3). The MCC beads containing bound proteins were regarded as bound fraction. The protein samples prepared from tobacco leaves were subjected to SDS-PAGE, followed by Commassie blue staining or Western blot analysis.

### Western Blot Analysis

The protein samples prepared from tobacco leaves were separated by SDS-PAGE using 10% acrylamide gel and transferred onto a polyvinylidene fluoride membrane. The membrane containing protein samples was incubated with 1 x TBS-T (Tris-buffered saline with 0.1% (v/v) Tween 20) solution containing 6% (w/v) skim milk for 30 min. The membrane was then incubated with an anti-CBM3 antibody (1:5,000 dilution) (BioApplications Inc., Korea) at 4°C for 4 h. After washing with 1 x TBS-T three times, the membrane was incubated with an anti-rabbit secondary antibody conjugated with horseradish peroxidase (1:5,000 dilution) (Bethyl Laboratories Inc.) at 4°C for 4 h. Finally, after washing with 1 x TBS-T three times, the membrane was immersed in ECL reagents (Thermo Fisher Scientific Inc.) and the chemiluminescence images were captured using the ChemiDoc^TM^ XRS+ imaging system (Bio-Rad Laboratories, Inc.).

### Endoglucanase Activity of GtCel12A-CBM3 Expressed in the *N. benthamiana* Leaves

The endoglucanase activity of BiP-M-bdSUMO-GtCel12A-CBM3-HDEL immobilized on MCC beads was directly tested as described previously (Oh et al., 2019). The purified enzyme (5 μg) was incubated with 0.5% CMC substrate at indicated pH and temperatures for 2 h. The buffer conditions for GtCel12A enzyme reaction at different pH were the same as those in the previous study (Oh et al., 2019). All the reactions were performed three times using purified proteins prepared from different tobacco leaves (biological replicates) and each reaction was performed in triplicate per plate (technical replicates). During the enzymatic reaction, samples were occasionally mixed by inversion, considering the spontaneous sedimentation of MCC beads. After the reaction, the samples were boiled for 1 min for enzyme inactivation. Then, 100 μL of each sample was incubated with 300 μL of 0.5% [w/v] 3,5-dinitrosalicylic acid (DNS) solution at 100°C for 5 min to detect reducing sugars; the amount of reducing sugars was quantified using a spectrophotometer (Multiskan EX, Thermo Fisher Scientific, Vantaa, Finland) at 550 nm. One unit (U) is defined as the amount of enzyme required to produce 1 μmol of reducing sugars per min.

## Results and Discussion

### Construct Design for High-Level GtCel12A-CBM3 Expression in Plants

**Figure 1:**
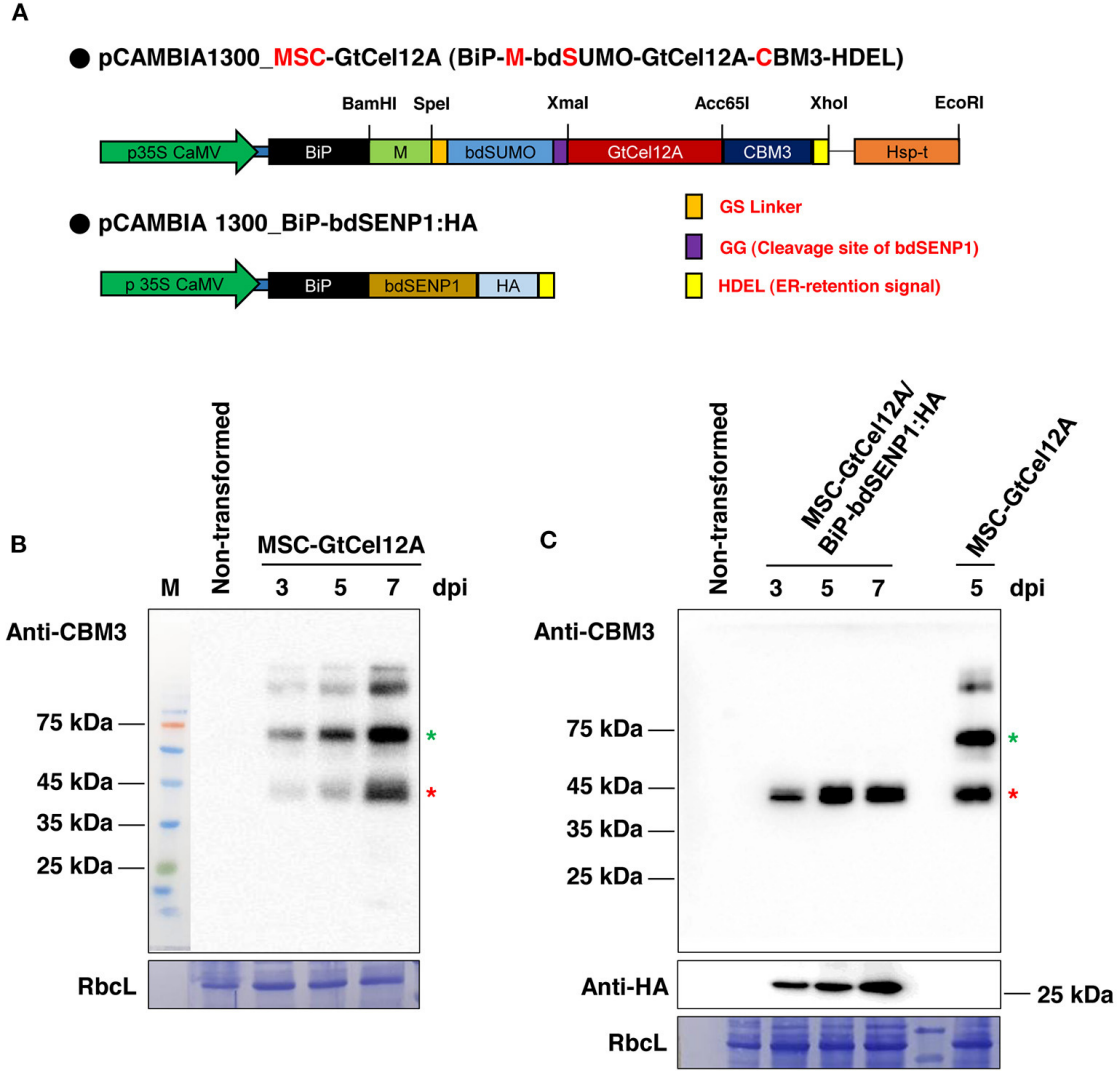
Transient expression of *MSC-GtCel12A* in *Nicotiana benthamiana*. **(A)** Schematic representation of *MSC-GtCel12A* and *BiP-bdSENP1:HA*. BiP, the signal sequence of BiP; M, the extracellular domain (amino acid residues 231–290) of human protein tyrosine phosphatase receptor type C; bdSUMO, the SUMO domain of *Brachypodium distachyon*; CBM3, the cellulose-binding module 3 of *Clostridium thermocellum*; HDEL, ER retention signal. **(B,C)** Expression of *MSC-GtCel12A* in *N. benthamiana*. The leaf tissues of *N. benthamiana* were infiltrated using a syringe with *A. tumefaciens* harboring the indicated constructs. *A. tumefaciens* harboring *P38* were co-infiltrated in every agroinfiltration. The transformed leaves were harvested 3, 5, and 7 days post-infiltration (dpi), followed by total protein extract preparation. Total protein extracts (20 μg) were analyzed by western blot analysis using anti-CBM3 or anti-HA antibodies. RbcL, large subunit of the Rubisco complex stained with Coomassie Brilliant Blue (CBB).

For an upscaled GtCel12A endoglucanase production in the *N. benthamiana* leaves, we designed the expression cassette MSC-GtCel12A in the binary vector pCAMBIA1300 (Figure 1A). To accumulate the target protein GtCel12A in the ER, we incorporated the BiP signal sequence and the ER retention signal HDEL into the N- and C-terminal parts, respectively, of the expression cassette. We also incorporated the M-domain of the human receptor-type tyrosine-protein phosphatase C, containing four N-glycosylation sites, as it reportedly enhances the expression of ER-localized proteins remarkably in plants (Kang et al., 2018). After the M domain, we added the bdSUMO (*Brachypodium distachyon* Small Ubiquitin-like Modifier) and a GG (Gly-Gly) motif, providing the binding site for the protease bdSENP1 (*Brachypodium distachyon* Sentrin/SUMO-specific protease-1) for cleavage immediately after the GG motif, thereby helping to achieve the removal of the unnecessary BiP signal peptide, M domain, and bdSUMO after the production of GtCel12A in the ER (Figure 1A) (Islam et al., 2019, 2020). After incorporating the GtCel12A coding sequence, we added the coding sequence of CBM3 (family 3 cellulose-binding module) that irreversibly binds to MCC beads and has been previously used as an affinity tag (Islam et al., 2019).

### GtCel12A-CBM3 Was Highly and Exclusively Expressed in the Soluble Fraction in Plants

**Figure 2:**
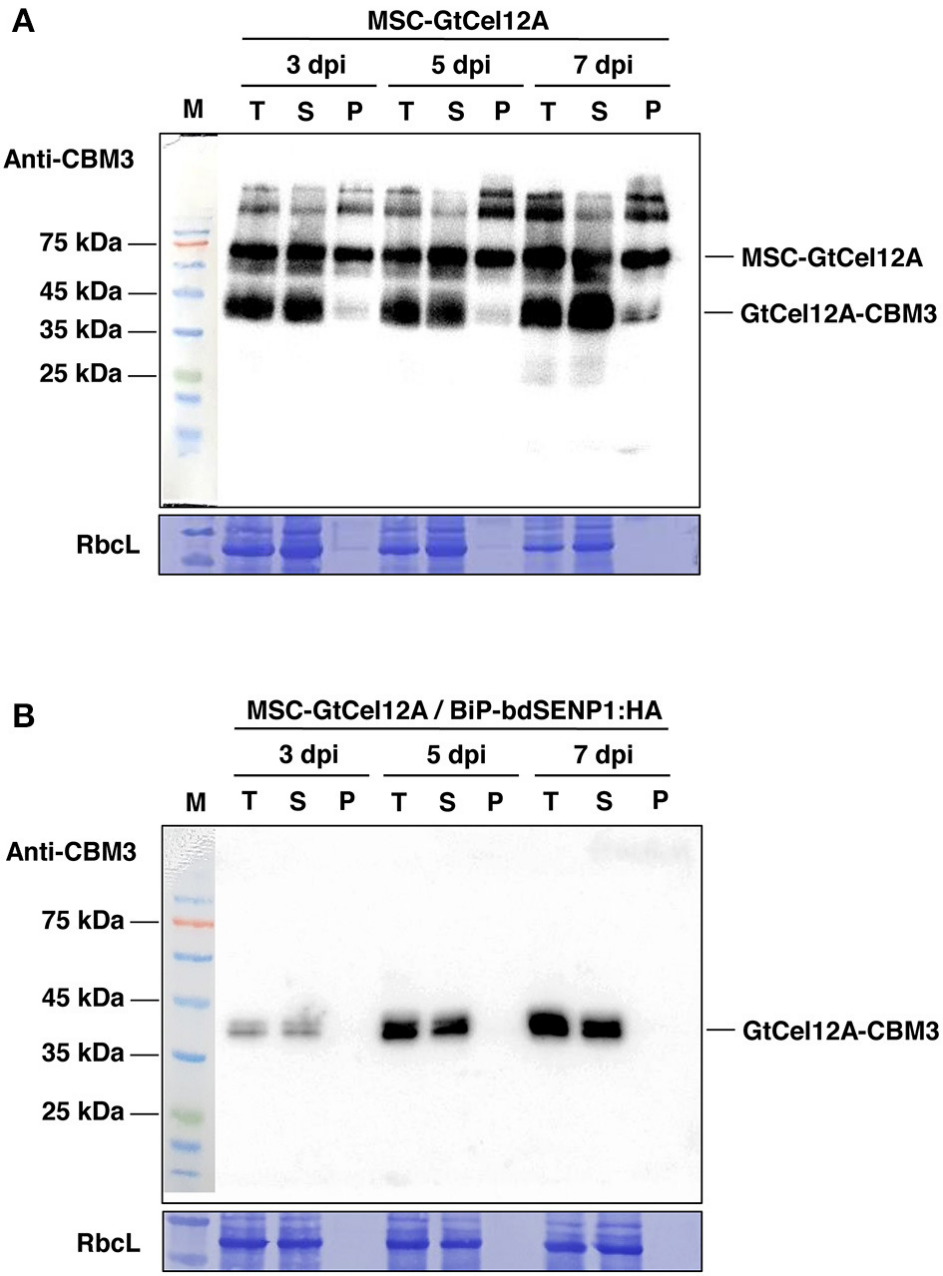
Solubility of GtCel12A-CBM3 produced in *N. benthamiana*. **(A,B)** Fractionation of total protein extracts prepared from the leaf tissues transformed with the indicated constructs. Total protein extracts prepared from 0.2 g of leaves were subjected to centrifugation at 13.000 rpm for 10 min. Next, supernatant (soluble) and pellet fractions were collected separately for western blot analysis using the anti-CBM3 antibody. T, total fraction; S, soluble fraction; P, pellet fraction; RbcL, large subunit of the Rubisco complex stained with Coomassie Brilliant Blue (CBB).

Next, we transformed the construct *MSC-GtCel12A* alone or together with *BiP-bdSENP1-HA* into the *N. benthamiana* leaves using agroinfiltration (Figures 1B,C) (Islam et al., 2019; Razzak et al., 2020). At 3, 5, and 7 days post infiltration (dpi), we isolated total protein extracts from the transformed leaves and analyzed them by western blotting using an anti-CBM3 antibody. The amount of the expressed proteins increased gradually with time (Figures 1B,C). In the absence of BiP-bdSENP1-HA, MSC-GtCel12A was present at multiple locations in the SDS-PAGE gel (Figure 1B). The upper two bands above the full-length MSC-GtCel12A (indicated with a green asterisk) are regarded as glycosylated forms caused by the presence of the highly glycosylated M-domain and were not further investigated. Besides these upper bands, MSC-GtCel12A was present in the cleaved (red asterisk) and intact form (green asterisk). Intriguingly, the size of the cleaved form was equivalent to that of the fragment GtCel12A-CBM3-HDEL, in which the N-terminal BiP signal sequence, M domain, and bdSUMO were removed by the ER-localized bdSENP1, which was produced by co-infiltration with *BiP-bdSENP1-HA* (Figure 1C). The predicted molecular weight of GtCel12A-CBM3-HDEL is ~43.45 kilodaltons (kDa), which is in good agreement with the size of the cleaved form (Figure 1C). Therefore, it is likely that MSC-GtCel12A could be spontaneously cleaved to produce a GtCel12A-CBM3 fusion protein through an unknown mechanism, even in the absence of bdSENP1. Next, we addressed the solubility of MSC-GtCel12A, which is crucial for the efficient purification and functionality of the produced enzymes (Figure 2). To perform this step, total protein extracts from the transformed plants were separated into soluble and pellet fractions by centrifugation. The full-length MSC-GtCel12A was present in both fractions (Figure 2A). However, the fragment GtCel12A-CBM3, the final product in this study, was present exclusively in the soluble fraction, regardless of the presence of the ER-localized bdSENP1, which is a desirable feature for the affinity purification and functionality of the produced enzymes (Figures 2A,B). These results also suggest that, although the M domain and SUMO domain play crucial roles in protein production by improving translational efficiency and removing domains not required for the functionality of the produced proteins, respectively, they may adversely affect the solubility of target proteins.

### MCC Bead-Immobilized GtCel12A-CBM3 Displayed Efficient Endoglucanase Activity on CMC

**Figure 3:**
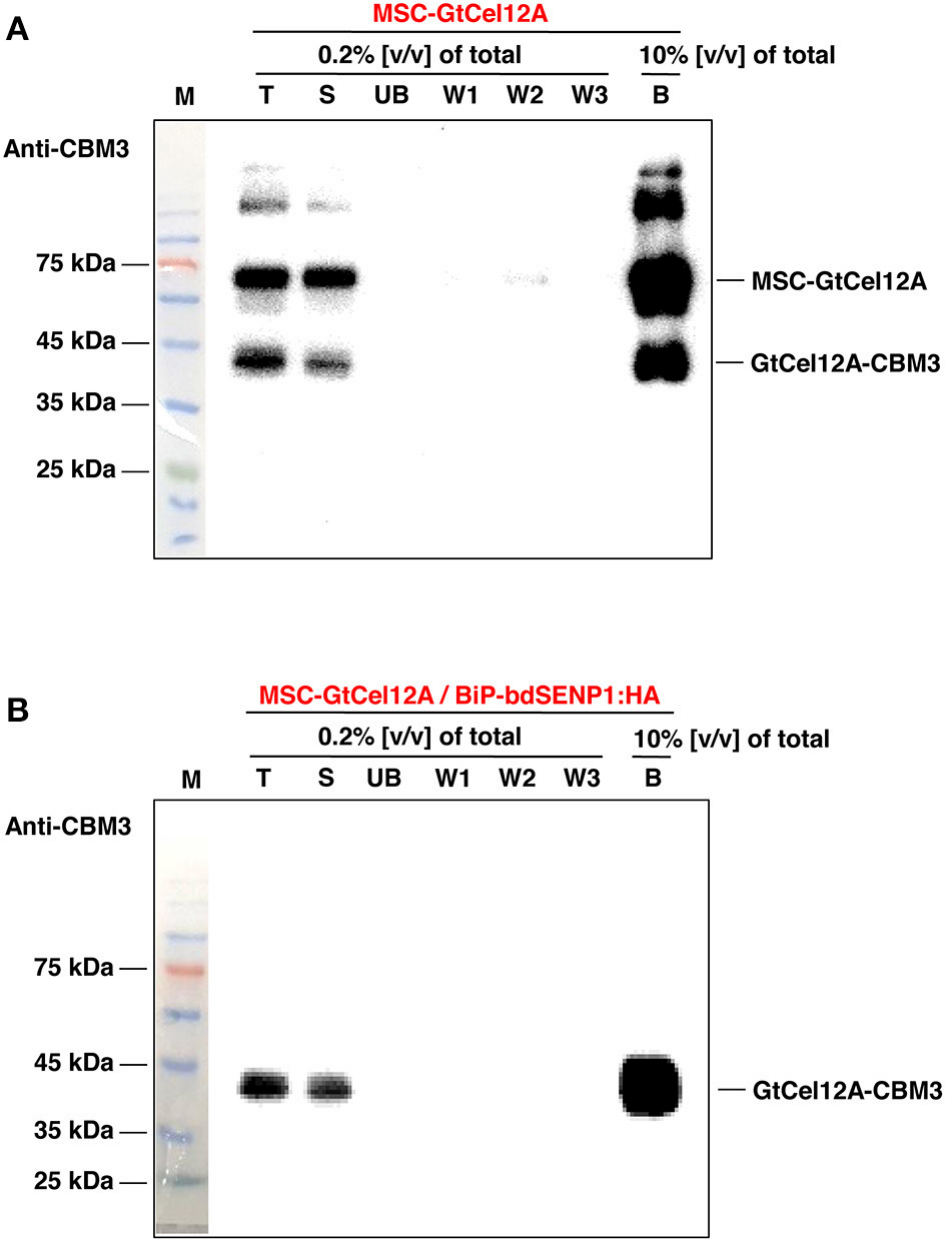
Purification of MSC-GtCel12A from the *N. benthamiana* leaf extracts. **(A,B)** To purify CBM3-fused GtCel12A, 4 grams of leaves harvested 7 days after agro-infiltration were used as described in the Materials and Methods. Total protein extracts were incubated with MCC beads. After centrifugation, the supernatant fraction was collected as unbound (UB) fraction. Next, the MCC beads were washed three times with a wash buffer. Finally, MCC bead-bound GtCel12A was eluted by boiling with protein sample buffer. T, total protein extracts; S, soluble fraction used to incubate with the MCC beads; UB, unbound fraction; B, MCC bead-bound fraction.

To examine the endoglucanase activity of the GtCel12A produced in *N. benthamiana*, the GtCel12A-CBM3 fusion protein was purified using MCC beads, which exhibit a specific affinity for CBM3 (Figure 3 and Supplementary Figure 1) (Islam et al., 2019). The protein yield of GtCel12A-CBM3 was estimated to be ~50 mg/kg fresh mass of *N. benthamiana* leaves at approximately >90% purity, based on the SDS-PAGE analysis (Supplementary Figure 2). In regard to the recovery of produced proteins, we consider that more than 95% of expressed GtCel12A-CBM3 proteins were immobilized on MCC beads, because GtCel12A-CBM3 was exclusively present in soluble fraction (Figure 2B) which was used for purification, and no GtCel12A-CBM3 was detected in unbound (UB) fraction (Figure 3B). In this study, we intended to develop a platform using GtCel12A immobilized on MCC beads. The binding between CBM3 and MCC beads is almost irreversible, thereby hindering the release of target proteins from the beads (Pinto et al., 2004; You and Zhang, 2013). However, this tight binding can be considered beneficial for not only the efficient capture and recovery of target proteins but also the repeated utilization of target proteins. Previous studies have indicated that industrial enzymes such as carbonic anhydrases from different origins exhibit not only efficient enzyme activity but also high endurability, thereby allowing multiple reuses of produced enzymes (Kumari et al., 2020; Razzak et al., 2020).

**Figure 4:**
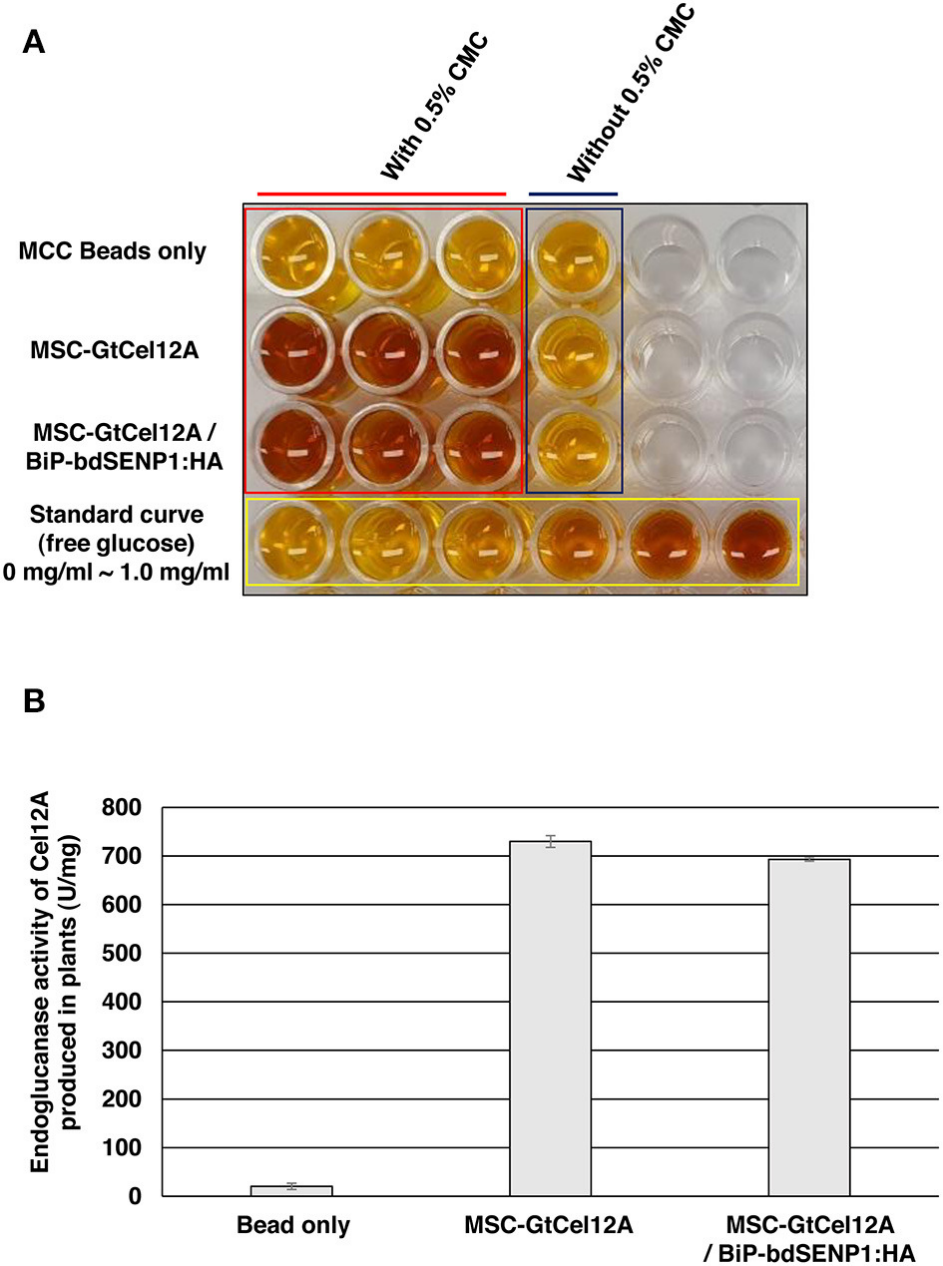
GtCel12A-CBM3 immobilized on MCC beads harbors endoglucanase activity. **(A)** The endoglucanase activity of GtCel12A-CBM3 immobilized on MCC beads was determined by reducing sugar content measurement using dinitrosalicylic acid (DNS) assay. The endoglucanase activity is shown by the change in DNS color. **(B)** Quantification of specific GtCel12A-CBM3 activity. One unit (U) is defined as the amount of GtCel12A-CBM3 that can release 1 μmol of glucose from CMC per min. All the reactions were performed three times (biological replicates) and each reaction was performed in triplicate per plate (technical replicates). Data represent means (*n* = 3) ± standard deviation in which n represents biological replicates.

The MCC bead-immobilized GtCel12A-CBM3 was directly tested for its endoglucanase activity on the CMC substrate at 50°C and pH 3.0, as described previously (Figure 4) (Oh et al., 2019). In the absence of BiP-bdSENP1-HA, the purified MSC-GtCel12A were present in both the full-length and GtCel12A-CBM3 forms that were both used in the enzyme assay (Figure 1). The endoglucanase activity was measured by a DNS assay, which has been widely applied for cellulose-derived reducing sugar content measurements (Song et al., 2016). Notably, the MCC bead-immobilized GtCel12A-CBM3 possessed high endoglucanase activity as indicated by the change in DNS color (Figure 4A) and increased amounts of reducing sugars after enzyme reaction (Figure 4B), suggesting the possibility that the GtCel12A-CBM3 fusion protein could be used in multiple rounds of enzyme reactions for cellulose decomposition, thereby potentially reducing the bioethanol production cost.

**Figure 5:**
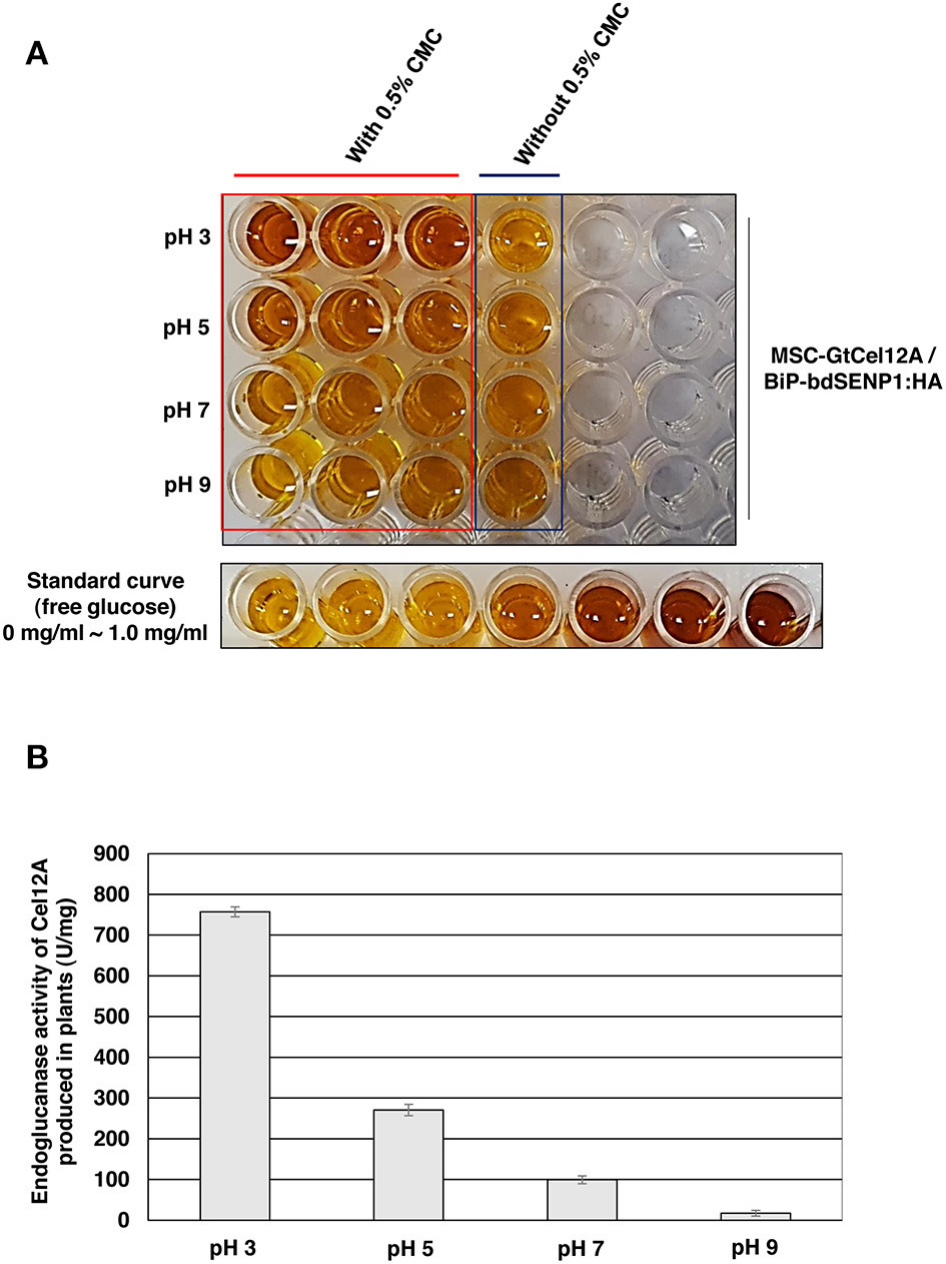
GtCel12A-CBM3 immobilized on MCC beads exhibits efficient endoglucanase activity at pH 3.0**. (A)** Endoglucanase activity of GtCel12A-CBM3 immobilized on MCC beads was determined at indicated pH by measuring the reducing sugar content using dinitrosalicylic acid (DNS) assay. The enzyme reaction was performed at 50°C. The endoglucanase activity is shown by the change in DNS color. **(B)** Quantification of specific GtCel12A-CBM3 activity. One unit (U) is defined as the amount of GtCel12A-CBM3 that can release 1 μmol of glucose from CMC per min. All the reactions were performed three times (biological replicates) and each reaction was performed in triplicate per plate (technical replicates). Data represent means (*n* = 3) ± standard deviation in which n represents biological replicates.

**Figure 6:**
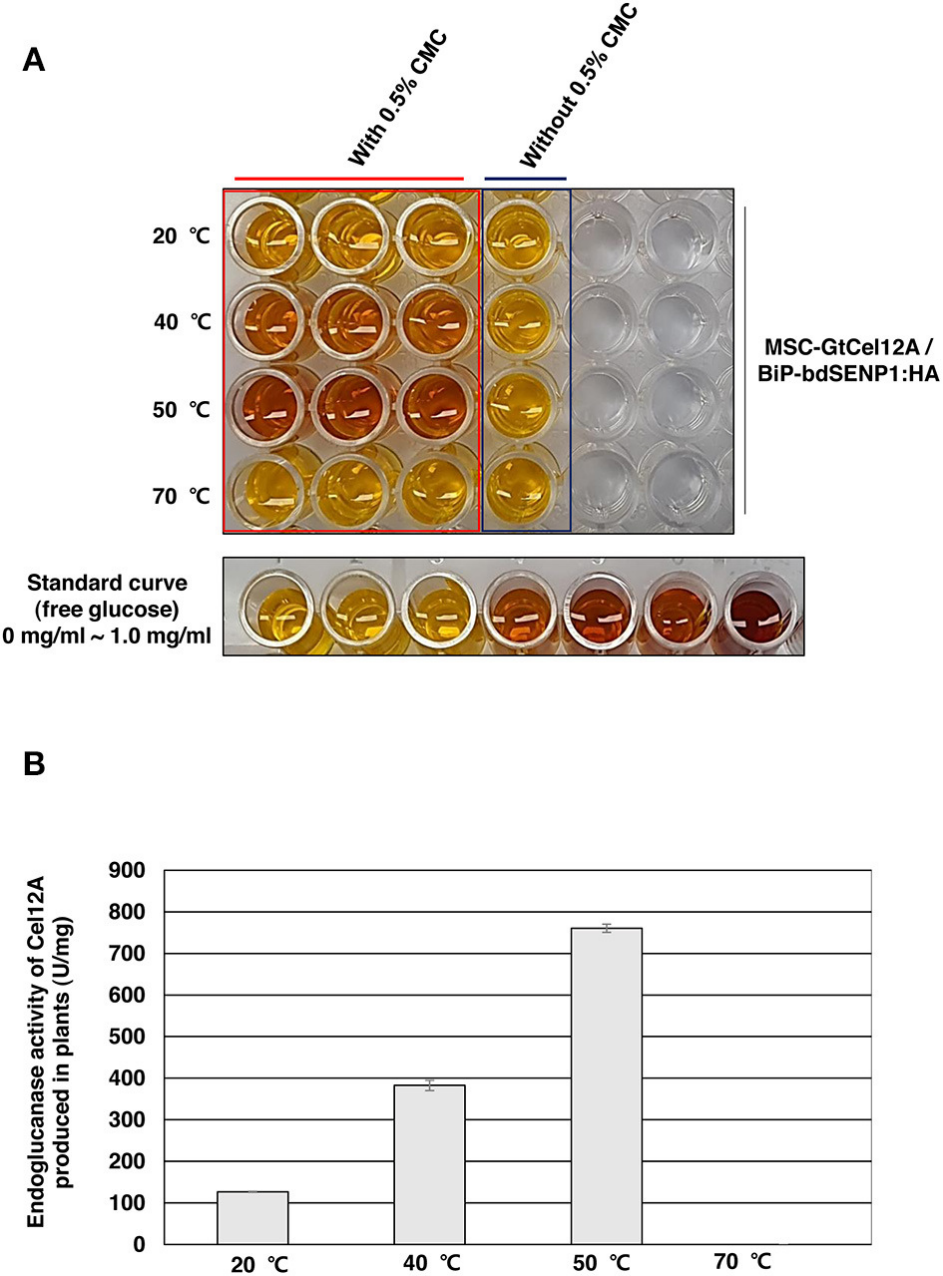
GtCel12A-CBM3 immobilized on MCC beads exhibits efficient endoglucanase activity at 50°C**. (A)** Endoglucanase activity of GtCel12A-CBM3 immobilized on MCC beads was determined at indicated temperatures by measuring the reducing sugar content using dinitrosalicylic acid (DNS) assay. The enzyme reaction was performed at pH 3.0. The endoglucanase activity is shown by the change in DNS color. **(B)** Quantification of specific GtCel12A-CBM3 activity. One unit (U) is defined as the amount of GtCel12A-CBM3 that can release 1 μmol of glucose from CMC per min. All the reactions were performed three times (biological replicates) and each reaction was performed in triplicate per plate (technical replicates). Data represent means (*n* = 3) ± standard deviation in which n represents biological replicates.

According to previous studies, GtCel12A produced from *P. pastoris* displayed efficient endoglucanase activity over a range of highly acidic conditions (pH 2.0–4.0) whereas that produced from *Aspergillus niger* exhibited an optimal activity at pH 4.5 (Miotto et al., 2014; Oh et al., 2019). To address the possibility that the optimal enzyme activity of GtCel12A-CBM3 produced from *N. benthamiana* and immobilized on MCC beads is different from that of GtCel12A produced from *P. pastoris* or *A. niger*, we tested the enzyme activity of GtCel12A-CBM3 immobilized on MCC beads at different ranges of pH and temperatures (Figures 5, 6). In these experiments, we removed the N-terminal M and SUMO domains by co-expression of *BiP-bdSENP1-HA*. Consistent with the previous result from Oh et al. (2019), our result showed that GtCel12A produced from *N. benthamiana* exhibited optimal enzyme activity at pH 3 (Figure 5) and 50°C (Figure 6).

In conclusion, in this study we developed a plant-based platform to produce a potent cellulase GtCel12A. GtCel12A-CBM3 produced from *N. benthamiana* was markedly soluble without any degradation. Moreover, GtCel12A-CBM3 exhibited efficient endoglucanase activity even when immobilized on MCC beads, enabling the reuse of GtCel12A enzyme for cellulose hydrolysis. There have been several previous attempts to produce cellulases in plants. For example, Dai et al. (2000) and Jin et al. (2003) produced chloroplast-targeted versions of *Acidothermus cellulolyticus* endoglucanase E1 in tobacco. In another study, Kim et al. (2010) also tried to accumulate *Thermotoga maritima* endoglucanase Cel5A in the chloroplasts of transgenic tobacco. All those studies indicate that both the expression level and the import efficiency of cellulases into chloroplasts are remarkably affected by the identity and length of transit peptides that might ultimately contribute to the endoglucanase activity of crude extracts from transgenic plants (Dai et al., 2000; Jin et al., 2003; Kim et al., 2010). In this study, we aimed to accumulate GtCel12A in the ER. Unlike protein import into chloroplasts, protein translocation across the ER membrane occurs co-translationally (Nyathi et al., 2013). Further, ER luminal proteins can be accumulated in the ER by simply adding an ER retention signal at the C-terminus (Figure 1A). Moreover, the M-domain of the human receptor-type tyrosine-protein phosphatase C, a target of N-glycosylation in the ER, might increase the translational level of GtCel12A (Kang et al., 2018). The activity of GtCel12A produced in the ER of tobacco might be affected by the plant-specific post-translational modifications in the ER (Van Eerde et al., 2020). We consistently obtained the specific activity of plant-produced GtCel12A (~750 U/mg), which was lower than that of GtCel12A produced from *P. pastoris* (~1,129 U/mg) (Oh et al., 2019). We consider that the efficient recovery and reuse in multiple rounds of enzyme reactions through immobilization of GtCel12A-CBM3 on MCC beads may aid in overcoming this limitation. Further improvement of GtCel12A activity through directed evolution may also help to develop an improved platform for cellulose degradation (Contreras et al., 2020). Finally, the strategies to scale up the production of GtCel12A in *N. benthamiana* will be necessary for sustainable application of this enzyme in cellulose degradation. In this study, to enhance the expression of GtCel12A-CBM3, the double Cauliflower mosaic virus 35S promoter, a strong 5′-UTR (Kim et al., 2014), HSP transcriptional terminator (Nagaya et al., 2010), and M-domain of the human receptor-type tyrosine-protein phosphatase C (Kang et al., 2018) were incorporated into expression cassettes. Along with these sequence elements, it will be necessary to develop the practical ways to achieve massive production of GtCel12A-CBM3. These may include the establishment of stable overexpression lines which can be easily scaled up and innovative viral vectors (Yamamoto et al., 2018). In addition, to enhance the transfection efficiency of *GtCel12A-CBM3, Agrobacterium* spray-based transfection process can be used because in this expression system, the viral replicons required for cell-to-cell movement incredibly increased the transfection levels to about 90% of leaf cells of *Nicotiana* plants (Hahn et al., 2015). Furthermore, plants provide an additional promising platform known as chloroplast transformation, which will possibly contribute to the high-level expression of high-value proteins such as industrial enzymes including GtCel12A and biopharmaceuticals (Verma and Daniell, 2007; Agrawal et al., 2011; Daniell et al., 2019).

## Data Availability Statement

The original contributions presented in the study are included in the article/Supplementary Material, further inquiries can be directed to the corresponding author.

## Author Contributions

DL conceived this project. KG performed most of the experiments. YS and H-JB contributed to measuring the endoglucanase activity of GtCel12A. IH participated in the discussion. KG and DL wrote the manuscript. All authors contributed to the article and approved the submitted version.

## Conflict of Interest

The authors declare that the research was conducted in the absence of any commercial or financial relationships that could be construed as a potential conflict of interest.

## References

[B1] Agrawal P. Verma D. Daniell H. (2011). Expression of Trichoderma reesei beta-mannanase in tobacco chloroplasts and its utilization in lignocellulosic woody biomass hydrolysis. PLoS ONE 6:e29302. 10.1371/journal.pone.0029302 22216240 PMC3247253

[B2] Ali N. Zhang Q. Liu Z. Y. Li F. L. Lu M. Fang X. C. (2020). Correction to: emerging technologies for the pretreatment of lignocellulosic materials for bio-based products. Appl. Microbiol. Biotechnol. 104:5159. 10.1007/s00253-020-10629-5 32337629

[B3] Barbosa F. C. Silvello M. A. Goldbeck R. (2020). Cellulase and oxidative enzymes: new approaches, challenges and perspectives on cellulose degradation for bioethanol production. Biotechnol. Lett. 42, 875–884. 10.1007/s10529-020-02875-4 32239348

[B4] Behera S. S. Ray R. C. (2016). Solid state fermentation for production of microbial cellulases: recent advances and improvement strategies. Int. J. Biol. Macromol. 86, 656–669. 10.1016/j.ijbiomac.2015.10.090 26601764

[B5] Bischof R. H. Ramoni J. Seiboth B. (2016). Cellulases and beyond: the first 70 years of the enzyme producer Trichoderma reesei. Microb. Cell Fact. 15:106. 10.1186/s12934-016-0507-6 27287427 PMC4902900

[B6] Buyel J. F. Twyman R. M. Fischer R. (2017). Very-large-scale production of antibodies in plants: the biologization of manufacturing. Biotechnol. Adv. 35, 458–465. 10.1016/j.biotechadv.2017.03.011 28347720

[B7] Cho E. J. Trinh L. T. P. Song Y. Lee Y. G. Bae H. J. (2020). Bioconversion of biomass waste into high value chemicals. Bioresour. Technol. 298:122386. 10.1016/j.biortech.2019.122386 31740245

[B8] Contreras F. Pramanik S. Rozhkova A. M. Zorov I. N. Korotkova O. Sinitsyn A. P.. (2020). Engineering robust cellulases for tailored lignocellulosic degradation cocktails. Int. J. Mol. Sci. 21:51589. 10.3390/ijms21051589 32111065 PMC7084875

[B9] Dai Z. Hooker B. S. Anderson D. B. Thomas S. R. (2000). Expression of Acidothermus cellulolyticus endoglucanase E1 in transgenic tobacco: biochemical characteristics and physiological effects. Transgenic Res. 9, 43–54. 10.1023/A:1008922404834 10853268

[B10] Daniell H. Rai V. Xiao Y. H. (2019). Cold chain and virus-free oral polio booster vaccine made in lettuce chloroplasts confers protection against all three poliovirus serotypes. Plant Biotechnol. J. 17, 1357–1368. 10.1111/pbi.13060 30575284 PMC6576100

[B11] Fatma S. Hameed A. Noman M. Ahmed T. Shahid M. Tariq M.. (2018). Lignocellulosic biomass: a sustainable bioenergy source for the future. Protein Pept. Lett. 25, 148–163. 10.2174/0929866525666180122144504 29359659

[B12] Garvey M. Klinger J. Klose H. Fischer R. Commandeur U. (2014). Expression of recombinant cellulase Cel5A from Trichoderma reesei in tobacco plants. J. Vis. Exp. 13:e51711. 10.3791/51711 24962636 PMC4189538

[B13] Garvey M. Klose H. Fischer R. Lambertz C. Commandeur U. (2013). Cellulases for biomass degradation: comparing recombinant cellulase expression platforms. Trends Biotechnol. 31, 581–593. 10.1016/j.tibtech.2013.06.006 23910542

[B14] Hahn S. Giritch A. Bartels D. Bortesi L. Gleba Y. (2015). A novel and fully scalable Agrobacterium spray-based process for manufacturing cellulases and other cost-sensitive proteins in plants. Plant Biotechnol. J. 13, 708–716. 10.1111/pbi.12299 25470212

[B15] Islam M. R. Choi S. Muthamilselvan T. Shin K. Hwang I. (2020). *In vivo* removal of N-terminal fusion domains from recombinant target proteins produced in *Nicotiana benthamiana*. Front. Plant Sci. 11:440. 10.3389/fpls.2020.00440 32328082 PMC7160244

[B16] Islam M. R. Kwak J. W. Lee J. S. Hong S. W. Khan M. R. I. Lee Y.. (2019). Cost-effective production of tag-less recombinant protein in Nicotiana benthamiana. Plant Biotechnol. J. 17, 1094–1105. 10.1111/pbi.13040 30468023 PMC6523591

[B17] Jeswani H. K. Chilvers A. Azapagic A. (2020). Environmental sustainability of biofuels: a review. Proc. Math. Phys. Eng. Sci. 476:20200351. 10.1098/rspa.2020.0351 33363439 PMC7735313

[B18] Jin R. G. Richter S. Zhong R. Lamppa G. K. (2003). Expression and import of an active cellulase from a thermophilic bacterium into the chloroplast both *in vitro* and *in vivo*. Plant Mol. Biol. 51, 493–507. 10.1023/A:1022354124741 12650616

[B19] Kang H. Park Y. Lee Y. Yoo Y. J. Hwang I. (2018). Fusion of a highly N-glycosylated polypeptide increases the expression of ER-localized proteins in plants. Sci. Rep. 8:4612. 10.1038/s41598-018-22860-2 29545574 PMC5854594

[B20] Kim S. Lee D. S. Choi I. S. Ahn S. J. Kim Y. H. Bae H. J. (2010). Arabidopsis thaliana Rubisco small subunit transit peptide increases the accumulation of Thermotoga maritima endoglucanase Cel5A in chloroplasts of transgenic tobacco plants. Transgenic Res. 19, 489–497. 10.1007/s11248-009-9330-8 19851881

[B21] Kim Y. Lee G. Jeon E. Sohn E. J. Lee Y. Kang H.. (2014). The immediate upstream region of the 5 '-UTR from the AUG start codon has a pronounced effect on the translational efficiency in Arabidopsis thaliana. Nucleic Acids Res. 42, 485–498. 10.1093/nar/gkt864 24084084 PMC3874180

[B22] Knodler M. Buyel J. F. (2021). Plant-made immunotoxin building blocks: a roadmap for producing therapeutic antibody-toxin fusions. Biotechnol. Adv. 47:107683. 10.1016/j.biotechadv.2020.107683 33373687

[B23] Komori T. Imayama T. Kato N. Ishida Y. Ueki J. Komari T. (2007). Current status of binary vectors and superbinary vectors. Plant Physiol. 145, 1155–1160. 10.1104/pp.107.105734 18056865 PMC2151727

[B24] Kumar G. Kim S. H. Lay C. H. Ponnusamy V. K. (2020). Recent developments on alternative fuels, energy and environment for sustainability. Bioresour. Technol. 317:124010. 10.1016/j.biortech.2020.124010 32822890

[B25] Kumari M. Lee J. Lee D. W. Hwang I. (2020). High-level production in a plant system of a thermostable carbonic anhydrase and its immobilization on microcrystalline cellulose beads for CO2 capture. Plant Cell Rep. 39, 1317–1329. 10.1007/s00299-020-02566-4 32651706

[B26] Lambertz C. Garvey M. Klinger J. Heesel D. Klose H. Fischer R.. (2014). Challenges and advances in the heterologous expression of cellulolytic enzymes: a review. Biotechnol. Biofuels 7:135. 10.1186/s13068-014-0135-5 25356086 PMC4212100

[B27] Lee D. W. Hwang I. (2018). Evolution and design principles of the diverse chloroplast transit peptides. Mol. Cells 41, 161–167. 10.14348/molcells.2018.0033 29487274 PMC5881089

[B28] Li X. H. Wang D. Zhou F. Yang H. J. Bhaskar R. Hu J. B.. (2010). Cloning and expression of a cellulase gene in the silkworm, Bombyx mori by improved Bac-to-Bac/BmNPV baculovirus expression system. Mol. Biol. Rep. 37, 3721–3728. 10.1007/s11033-010-0025-2 20195768

[B29] Liu H. Sun J. Chang J. S. Shukla P. (2018). Engineering microbes for direct fermentation of cellulose to bioethanol. Crit. Rev. Biotechnol. 38, 1089–1105. 10.1080/07388551.2018.1452891 29631429

[B30] Liu Y. Cruz-Morales P. Zargar A. Belcher M. S. Pang B. Englund E.. (2021). Biofuels for a sustainable future. Cell 184, 1636–1647. 10.1016/j.cell.2021.01.052 33639085

[B31] Maki M. Leung K. T. Qin W. (2009). The prospects of cellulase-producing bacteria for the bioconversion of lignocellulosic biomass. Int. J. Biol. Sci. 5, 500–516. 10.7150/ijbs.5.500 19680472 PMC2726447

[B32] Margolin E. Chapman R. Williamson A. L. Rybicki E. P. Meyers A. E. (2018). Production of complex viral glycoproteins in plants as vaccine immunogens. Plant Biotechnol. J. 16, 1531–1545. 10.1111/pbi.12963 29890031 PMC6097131

[B33] Margolin E. Crispin M. Meyers A. Chapman R. Rybicki E. P. (2020a). A roadmap for the molecular farming of viral glycoprotein vaccines: engineering glycosylation and glycosylation-directed folding. Front. Plant Sci. 11:609207. 10.3389/fpls.2020.609207 33343609 PMC7744475

[B34] Margolin E. Oh Y. J. Verbeek M. Naude J. Ponndorf D. Meshcheriakova Y. A.. (2020b). Co-expression of human calreticulin significantly improves the production of HIV gp140 and other viral glycoproteins in plants. Plant Biotechnol. J. 18, 2109–2117. 10.1111/pbi.13369 32096288 PMC7540014

[B35] Miotto L. S. De Rezende C. A. Bernardes A. Serpa V. I. Tsang A. Polikarpov I. (2014). The characterization of the endoglucanase Cel12A from Gloeophyllum trabeum reveals an enzyme highly active on beta-glucan. PLoS ONE 9:e108393. 10.1371/journal.pone.0108393 25251390 PMC4177221

[B36] Moon K. B. Park J. S. Park Y. I. Song I. J. Lee H. J. Cho H. S.. (2019). Development of systems for the production of plant-derived biopharmaceuticals. Plants 9:10030. 10.3390/plants9010030 31878277 PMC7020158

[B37] Muthamilselvan T. Kim J. S. Cheong G. Hwang I. (2019). Production of recombinant proteins through sequestration in chloroplasts: a strategy based on nuclear transformation and post-translational protein import. Plant Cell Rep. 38, 825–833. 10.1007/s00299-019-02431-z 31139894

[B38] Nagaya S. Kawamura K. Shinmyo A. Kato K. (2010). The HSP terminator of Arabidopsis thaliana increases gene expression in plant cells. Plant Cell Physiol. 51, 328–332. 10.1093/pcp/pcp188 20040586

[B39] Nyathi Y. Wilkinson B. M. Pool M. R. (2013). Co-translational targeting and translocation of proteins to the endoplasmic reticulum. Biochim. Biophys. Acta 1833, 2392–2402. 10.1016/j.bbamcr.2013.02.021 23481039

[B40] Obeng E. M. Adam S. N. N. Budiman C. Ongkudon C. M. Maas R. Jose J. (2017). Lignocellulases: a review of emerging and developing enzymes, systems, and practices. Bioresour. Bioprocess. 4:16. 10.1186/s40643-017-0146-8

[B41] Oh C. H. Park C. S. Lee Y. G. Song Y. Bae H. J. (2019). Characterization of acidic endoglucanase Cel12A from Gloeophyllum trabeum and its synergistic effects on hydrogen peroxide-acetic acid (HPAC)-pretreated lignocellulose. J. Wood Sci. 65:24. 10.1186/s10086-019-1803-7

[B42] Oh E. J. Jin Y. S. (2020). Engineering of Saccharomyces cerevisiae for efficient fermentation of cellulose. FEMS Yeast Res. 20:foz089. 10.1093/femsyr/foz089 31917414

[B43] Peterson R. Nevalainen H. (2012). Trichoderma reesei RUT-C30–thirty years of strain improvement. Microbiology 158, 58–68. 10.1099/mic.0.054031-0 21998163

[B44] Pinto R. Moreira S. Mota M. Gama M. (2004). Studies on the cellulose-binding domains adsorption to cellulose. Langmuir 20, 1409–1413. 10.1021/la035611u 15803726

[B45] Qu F. Ren T. Morris T. J. (2003). The coat protein of turnip crinkle virus suppresses posttranscriptional gene silencing at an early initiation step. J. Virol. 77, 511–522. 10.1128/JVI.77.1.511-522.2003 12477856 PMC140649

[B46] Razzak M. A. Lee D. W. Lee J. Hwang I. (2020). Overexpression and purification of Gracilariopsis chorda carbonic anhydrase (GcCAalpha3) in *Nicotiana benthamiana*, and its immobilization and use in CO2 hydration reactions. Front. Plant Sci. 11:563721. 10.3389/fpls.2020.563721 33329625 PMC7717956

[B47] Saroj P. Manasa P. Narasimhulu K. (2018). Characterization of thermophilic fungi producing extracellular lignocellulolytic enzymes for lignocellulosic hydrolysis under solid-state fermentation. Bioresour. Bioprocess. 5:31. 10.1186/s40643-018-0216-6

[B48] Schillberg S. Finnern R. (2021). Plant molecular farming for the production of valuable proteins - critical evaluation of achievements and future challenges. J. Plant Physiol. 258–259:153359. 10.1016/j.jplph.2020.153359 33460995

[B49] Song H. T. Gao Y. Yang Y. M. Xiao W. J. Liu S. H. Xia W. C.. (2016). Synergistic effect of cellulase and xylanase during hydrolysis of natural lignocellulosic substrates. Bioresour. Technol. 219, 710–715. 10.1016/j.biortech.2016.08.035 27560367

[B50] Srivastava R. K. Shetti N. P. Reddy K. R. Kwon E. E. Nadagouda M. N. Aminabhavi T. M. (2021). Biomass utilization and production of biofuels from carbon neutral materials. Environ. Pollut. 276:116731. 10.1016/j.envpol.2021.116731 33607352

[B51] Toor M. Kumar S. S. Malyan S. K. Bishnoi N. R. Mathimani T. Rajendran K.. (2020). An overview on bioethanol production from lignocellulosic feedstocks. Chemosphere 242:125080. 10.1016/j.chemosphere.2019.125080 31675581

[B52] Van Eerde A. Varnai A. Jameson J. K. Paruch L. Moen A. Anonsen J. H.. (2020). In-depth characterization of Trichoderma reesei cellobiohydrolase TrCel7A produced in Nicotiana benthamiana reveals limitations of cellulase production in plants by host-specific post-translational modifications. Plant Biotechnol. J. 18, 631–643. 10.1111/pbi.13227 31373133 PMC7004914

[B53] Verma D. Daniell H. (2007). Chloroplast vector systems for biotechnology applications. Plant Physiol. 145, 1129–1143. 10.1104/pp.107.106690 18056863 PMC2151729

[B54] Yamamoto T. Hoshikawa K. Ezura K. Okazawa R. Fujita S. Takaoka M.. (2018). Improvement of the transient expression system for production of recombinant proteins in plants. Sci. Rep. 8:4755. 10.1038/s41598-018-23024-y 29555968 PMC5859073

[B55] You C. Zhang Y. H. P. (2013). Self-assembly of synthetic metabolons through synthetic protein scaffolds: one-step purification, co-immobilization, and substrate channeling. ACS Synth. Biol. 2, 102–110. 10.1021/sb300068g 23656373

