# Supplementary material for: Production of Gloeophyllum trabeum Endoglucanase Cel12A in Nicotiana benthamiana for Cellulose Degradation
Source: Front Plant Sci. 2021 Jun 28;12:696199. doi: 10.3389/fpls.2021.696199 (PMC8273430; doi:10.3389/fpls.2021.696199)
Supplement: Supplementary file 1 [file Data_Sheet_1.PDF]

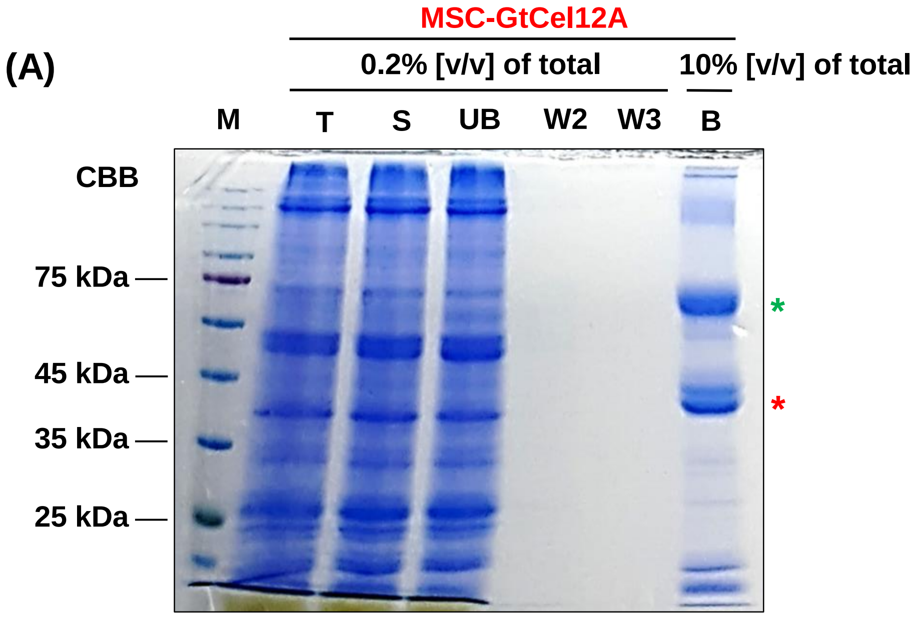

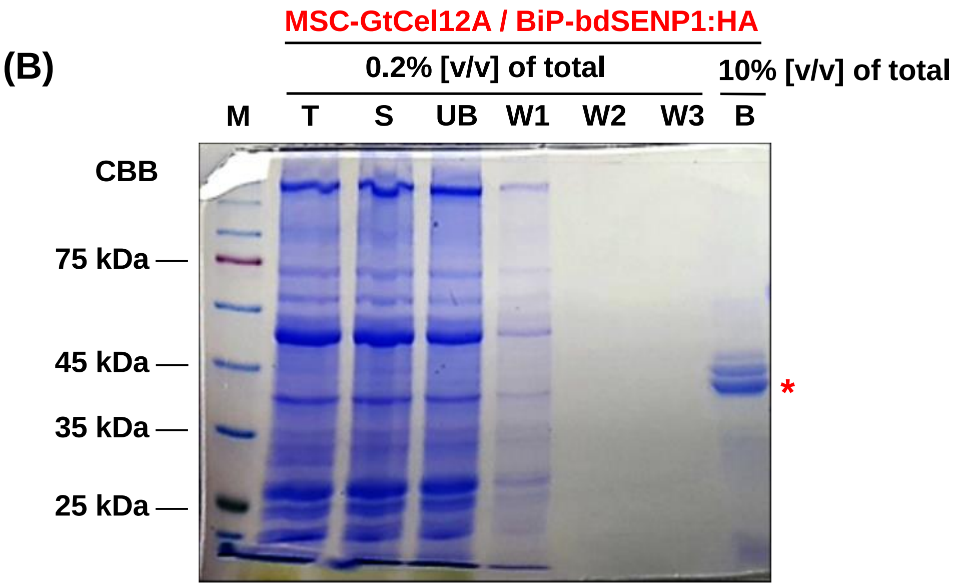

Supplementary Figure S1. Purification of MSC-GtCel12A from the *N. benthamiana* leaf extracts

(A and B) To purify CBM3-fused GtCel12A, 10 g of leaves harvested 7 days after agro-infiltration were used as described in the Materials and Methods. Total protein extracts were incubated with MCC beads. Unbound fractions (UB) were separately collected. Next, the MCC beads were washed three times with a wash buffer (50 mM Tris-HCl pH 7.5, 150 mM NaCl) (W1-W3). Finally, GtCel12A bound to MCC beads was eluted by boiling with protein sample buffer. Every fraction was loaded on SDS-PAGE, followed by staining with CBB. The images were captured using a digital camera. T, total protein extracts; S, soluble fraction used to incubate with the MCC beads; UB, unbound fraction; B, MCC bead-bound fraction.

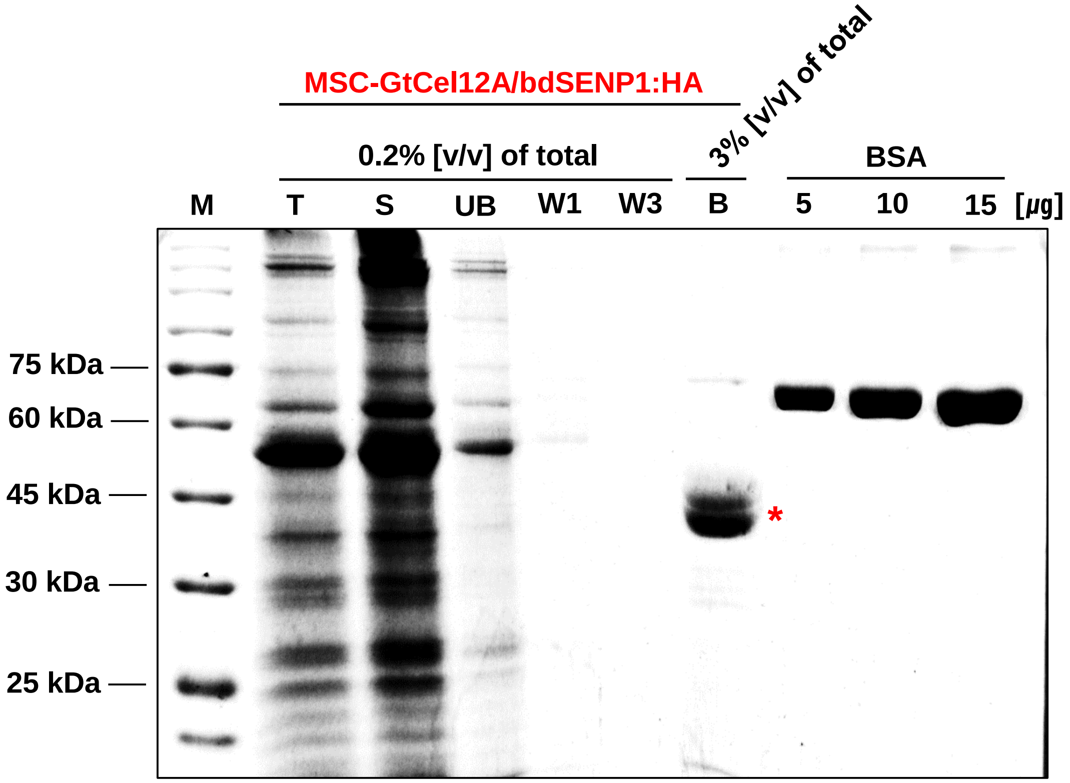

Supplementary Figure S2. Determination of the yield of purified GtCel12A-CBM3 from the *N. benthamiana* leaf extracts

To purify CBM3-fused GtCel12A, leaves (10 g) harvested 7 days after agro-infiltration were used as described in the Materials and Methods. Total protein extracts were incubated with microcrystalline cellulose (MCC) beads. Unbound (UB) fractions were separately collected. Next, the MCC beads were washed three times (W1-W3) with a wash buffer (50 mM Tris-HCl and 150 mM NaCl; pH 7.5). Finally, GtCel12A bound to MCC beads was eluted by boiling with protein sample buffer. Every fraction was loaded on SDS-PAGE, followed by staining with CBB. The image was captured using the ChemiDocTM XRS+ imaging system. The intensity of the purified band (B) and those of increasing amounts of BSA were measured using ImageJ software (National Institutes of Health, USA). Based on the BSA band intensities, the protein yield of GtCel12A-CBM3 was estimated to be approximately 50 mg/kg fresh weight (FW) of *N. benthamiana* leaves. T, total protein extracts; S, soluble fraction used to incubate with the MCC beads; UB, unbound fraction; B, MCC bead-bound fraction.
